# A Novel Prognostic Risk Model for Cervical Cancer Based on Immune Checkpoint HLA-G-Driven Differentially Expressed Genes

**DOI:** 10.3389/fimmu.2022.851622

**Published:** 2022-07-18

**Authors:** Hui-Hui Xu, Hui-Li Wang, Tong-Jin Xing, Xue-Quan Wang

**Affiliations:** ^1^ Medical Research Center, Taizhou Hospital of Zhejiang Province, Wenzhou Medical University, Linhai, China; ^2^ Key Laboratory of Minimally Invasive Techniques & Rapid Rehabilitation of Digestive System Tumor of Zhejiang Province, Taizhou Hospital of Zhejiang Province, Linhai, China; ^3^ Department of Burn, Taizhou Hospital of Zhejiang Province, Wenzhou Medical University, Linhai, China; ^4^ Department of Infectious Disease, Taizhou Hospital of Zhejiang Province, Wenzhou Medical University, Linhai, China; ^5^ Department of Radiation Oncology, Taizhou Hospital of Zhejiang Province, Wenzhou Medical University, Linhai, China

**Keywords:** HLA-G, cervical cancer, prognosis, prediction, mRNA signature, immune checkpoint

## Abstract

Human leukocyte antigen G (HLA-G) is a potential checkpoint molecule that plays a key role in cervical carcinogenesis. The purpose of this study was to construct and validate a prognostic risk model to predict the overall survival (OS) of cervical cancer patients, providing a reference for individualized clinical treatment that may lead to better clinical outcomes. HLA-G-driven differentially expressed genes (DEGs) were obtained from two cervical carcinoma cell lines, namely, SiHa and HeLa, with stable overexpression of HLA-G by RNA sequencing (RNA-seq). The biological functions of these HLA-G-driven DEGs were analysed by GO enrichment and KEGG pathway using the “clusterProfiler” package. The protein-protein interactions (PPIs) were assessed using the STRING database. The prognostic relevance of each DEG was evaluated by univariate Cox regression using the TCGA-CESC dataset. After the TCGA-CESC cohort was randomly divided into training set and testing set, and a prognostic risk model was constructed by LASSO and stepwise multivariate Cox regression analysis in training set and validated in testing set or in different types of cervical cancer set. The predictive ability of the prognostic risk model or nomogram was evaluated by a series of bioinformatics methods. A total of 1108 candidate HLA-G-driven DEGs, including 391 upregulated and 717 downregulated genes, were obtained and were enriched mostly in the ErbB pathway, steroid biosynthesis, and MAPK pathway. Then, an HLA-G-driven DEG signature consisting of the eight most important prognostic genes *CD46, LGALS9, PGM1, SPRY4, CACNB3, PLIN2, MSMO1*, and *DAGLB* was identified as a key predictor of cervical cancer. Multivariate Cox regression analysis showed that this signature is an independent risk factor for the overall survival of CESC patients. Kaplan-Meier survival analysis showed that the 5-year overall survival rate is 23.0% and 84.6% for the high-risk and low-risk patients, respectively (*P*<0.001). The receiver operating characteristic (ROC) curve of this prognostic model with an area under the curve (AUC) was 0.896 for 5 years, which was better than that of other clinical traits. This prognostic risk model was also successfully validated in different subtypes of cervical cancer, including the keratinizing squamous cell carcinoma, non-keratinizing squamous cell carcinoma, squamous cell neoplasms, non-squamous cell neoplasms set. Single-sample gene set enrichment (ssGSEA) algorithm and Tumor Immune Dysfunction and Exclusion (TIDE) analysis confirmed that this signature influence tumour microenvironment and immune checkpoint blockade. A nomogram that integrated risk score, age, clinical stage, histological grade, and pathological type was then built to predict the overall survival of CESC patients and evaluated by calibration curves, AUC, concordance index (C-index) and decision curve analysis (DCA). To summarize, we developed and validated a novel prognostic risk model for cervical cancer based on HLA-G-driven DEGs, and the prognostic signature showed great ability in predicting the overall survival of patients with cervical cancer.

## Introduction

Cervical cancer ranks as the fourth most prevalent cancer in the world (1). Persistent infection with human papillomavirus (HPV) has been identified as a necessary but not sufficient factor that leads to cervical cancer. More than 85% of the global cervical cancer burden occurs in less developed countries that lack organized screening and HPV vaccination programmes (2). Treatment depends on the disease extent at diagnosis and might involve surgical management for early-stage disease or adjuvant treatment or combination therapy for locally advanced disease. Survival prediction mainly depends on International Federation of Gynecology and Obstetrics (FIGO) stages. The expected 5-year survival rate remains poor (15% or less) for patients with recurrence or metastasis (stage IV) (3). Unfortunately, there are few effective therapeutic strategies specifically for recurrent or metastatic cervical cancer. Strikingly, immunotherapies hold promise for these patients (4). Thus, to identify novel biological markers, potential therapeutic targets and develop effective immunotherapy strategies are urgently needed in clinical settings.

Human leukocyte antigen G (HLA-G) is a tolerogenic nonclassical HLA-class I antigen with immunoinhibitory function. *HLA-G* gene polymorphism likely determines women at higher risk for cervical HPV infections and/or viral persistence (5). Ectopic expression of HLA-G molecule in cervical lesions helps tumour cells escape immunosurveillance by generating inhibitory signals (6). Increasing evidence suggests that HLA-G expression is strongly associated with high-grade tumour and poor prognosis in patients with cancer, especially lung cancer, colorectal cancer, cervical cancer (7). Based on the restricted tissue expression pattern and immunosuppressive functions of HLA-G molecule, many recent studies have consistently claimed that HLA-G could be a new immune checkpoint molecule in tumours (8–13). Our previous studies support that upregulation of HLA-G expression in the tumour microenvironment plays a key role in early cervical carcinogenesis (5, 6, 14). These results were in good agreement with results from other studies, and HLA-G may have significance in the early diagnosis of cervical malignant lesions (15–17). Notably, biomarkers concerning immune checkpoint blockade (ICB) therapy has become a promising strategy in cancer treatment. Therefore, it is urgent to understand the downstream signal pathway induced by HLA-G expression and the dynamic indicators to monitor the effect of immunotherapy after anti-HLA-G. However, its role in HLA-G-driven differentially expressed genes (DEGs) in tumour growth and advanced-stage cervical cancer remain unknown.

This study aimed to develop and validate a prognostic risk model by screening the immune checkpoint molecule HLA-G and its DEGs in cervical cancer based on RNA sequencing (RNA-seq) and the TCGA dataset by a series of bioinformatics methods.

## Materials and Methods

### Data Acquisition and Processing

**Figure 1:**
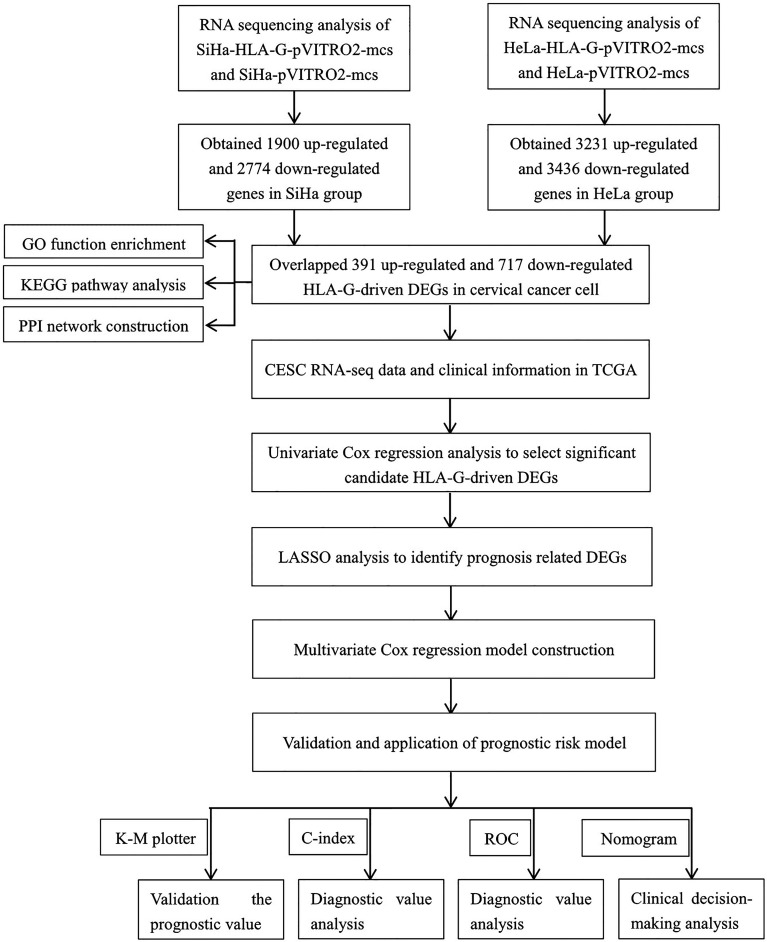
Flow diagram of this study.

The RNA-seq FPKM (fragments per kilobase of exon model per million mapped reads) data, Methylation450k data of patients with cervical cancer, and corresponding clinical data were obtained from the TCGA (https://portal.gdc.cancer.gov/). After removing normal samples, follow-up data and clinicopathological information were matched with the expression profiles. In the TCGA cervical squamous cell carcinoma (CESC) dataset, a total of 275 patients with a survival status and follow-up of more than 30 days were included (**Table 1**). The study design was illustrated in **Figure 1**.

**Table 1:** Clinical information of included CESC patients based on the overall survival in TCGA dataset.

Characteristics	Number			*P*-value
	Training set	Testing set	Entire set	
Cases	150	125	275	
**Age**				
< 45	91	76	167	0.9875
45-69	45	40	85
> 69	14	9	23
**Survival status**				
dead	35	37	72	0.5002
alive	115	88	203
**Histological type**				
Squamous Cell Neoplasms	121	108	229	0.5097
Non-Squamous Cell Neoplasms	29	17	46
**Keratinizing Classification**				
Keratinizing squamous cell carcinoma	26	22	48	0.9708
Non-keratinizing squamous cell carcinoma	57	49	106
**Pathologic_M**				
M0	49	52	101	0.3884
M1	6	5	11
MX	72	51	123
**Pathologic_N**				
N0	61	58	119	0.5933
N1	33	21	54
NX	33	31	64
**Pathologic_T**				
T1	72	56	128	0.3611
T2	31	34	65
T3	9	8	17
T4	4	6	10
TX	10	6	16
**Stage**				
Stage I	88	61	149	0.5606
Stage II	22	38	60
Stage III	26	13	39
Stage IV	10	11	21

### Cell Transfection Assays

The human cervical carcinoma cell lines SiHa and HeLa (ATCC, MD, USA) were cultured in DMEM supplemented with 10% heat-inactivated FBS (Gibco, Grand Island, USA). Cells were cultured at 37°C in a humidified 5% CO_2_ atmosphere. We constructed two cervical carcinoma cell lines that stably overexpressed HLA-G. Both SiHa and HeLa were transfected with the recombinant pVITRO2-hygro-mcs vector (*In vivo*Gen, USA) containing HLA-G cDNA or empty vector by Lipofectamine 3000 (InvitroGen, USA). The details of the transfection performance have been described previously (18).

### Cell Sequencing Data Analysis of the Transfectants

Total RNA was extracted from SiHa-pVITRO2-mcs, SiHa-HLA-G-pVITRO2-mcs, HeLa-pVITRO2-mcs and HeLa-HLA-G-pVITRO2-mcs cells. RNA sequencing was performed using the BGISEQ-500 platform (BGI, Wuhan, China). The details of the experimental process have been described in the previous study (19). The experimental process is based on the complete genomics sequencing technology and DNA NanoBalls (DNBs) technology for sequencing library construction and combined primer anchor synthesis (cPAS) for sequencing. Before downstream analysis, raw reads containing the adaptors sequences and low-quality reads were filtered out, and clean reads were mapped to reference genes using Bowtie2. Gene expression levels were quantified by the RSEM software package. Uniform manifold approximation and projection (UMAP) were performed to reduce the high-dimensional dataset to a three-dimensional (3D) space using the “umap” R package.

### Identification of HLA-G-Driven DEGs

The “DESeq2” R package was performed to screen differentially expressed genes induced by the HLA-G molecule in cervical carcinoma cells by comparing the whole transcriptome expression of SiHa-HLA-G-pVITRO2-mcs versus SiHa-pVITRO2-mcs cells and HeLa-HLA-G-pVITRO2-mcs versus HeLa-pVITRO2-mcs cells. The “ggplot2” and “pheatmap” packages were used to generate volcano and heat maps of HLA-G-driven DEGs. Meanwhile, an UpSet plot was drawn to screen out the upregulated and downregulated overlapping DEGs between the SiHa group and HeLa group (|Log_2_ Fold Change (FC) | > 0.5, *p*-value < 0.001) using the “UpSetR” R package.

### Gene Functional Enrichment Analyses

To investigate the biological functions of HLA-G-driven DEGs, functional enrichment was comprehensively detected by gene ontology (GO) terms enrichment and KEGG pathway using the “clusterProfiler” R package. GO enrichment was classified into three categories: biological processes (BP), cellular components (CC), and molecular functions (MF). GO enrichment and KEGG pathway were based on the threshold of *p*-value < 0.05, and the top ten enrichment items are illustrated in figures.

### Protein-Protein Interaction (PPI) Network Construction

Associations between these coding products of HLA-G-driven DEGs were constructed by the STRING database (https://string-db.org/). All *p*-values < 0.05 were considered significant. A PPI network was constructed based on these HLA-G-driven DEGs under the threshold of a minimum required interaction score > 0.4. Node genes that interact with HLA-G or prognostic model-related genes were retained and visualized using the Cytoscape open-source bioinformatics software platform (20).

### Evaluation of Methylation Prognostic Indicators

Methylation data of cervical cancer tissues matched with survival information of patients were used to assess the relationship between DNA methylation levels and overall survival (OS) status by univariate Cox regression analysis. In addition, eight signature-related gene DNA methylation statuses and their expression levels, clinical information and survival status were investigated through MEX. PRESS web sites (http://mexpress.be).

### Prognostic Risk Model Construction

The overlapping HLA-G-driven DEGs were analyzed by univariate Cox regression analysis using the “survival” package. Least absolute shrinkage and selection operator (LASSO) analysis was performed to select candidate DEGs with penalty parameter tuning adjusted by 10-fold cross validation using the “glmnet” package. To verify candidate DEGs and the risk model, the 275 enrolled patients in the TCGA-CESC dataset were randomly divided into training set (150 cases) and testing set (125 cases) using the “caret” package. The optimal prognostic signature was constructed based on the LASSO filtered candidate DEGs by the following stepwise multivariate Cox analysis in the training set using the “My.stepwise” package. Then, the testing set and entire set were used for validation. The risk score was calculated according to the following formula:


$$Risk\;score=\sum_{i=1}^{N}\text{βi}\left(\text{mRNAi}\right)*\text{expression}\left(\text{mRNAi}\right)$$

(N represents the number of gene signatures; βi represents the coefficients of each gene).

### Prognostic Signature for Cervical Cancer

CESC patients were divided into low-risk group and high-risk group according to the risk score using the surv_cutpoint function in the “survminer” package. Then, the risk score distribution, the survival status and survival time for each patient, as well as the heat map of the risk-related genes expression levels in each patient were plotted in the training set, testing set and entire set. Kaplan-Meier (KM) curves depicting overall survival were performed to investigate the survival difference between the above mentioned low-risk group and high-risk group. The predictive performance of the m6A risk signature for 1-, 3-, and 5-year OS was assessed by receiver operating characteristic (ROC) curves carried out using the “timeROC” package and concordance index (C-index) analyses were performed and validated in the training set, testing set, and entire set. The area under the curves (AUC) on the ROC were calculated.

### Validation of the Risk Score Signature for Subtypes of Cervical Cancer

The clinical data of cervical cancer subtypes were obtained from TCGA. Keratinizing squamous cell carcinoma (SCC) or non-keratinizing SCC, squamous cell neoplasms or non-squamous cell neoplasms were grouped and used to verify the corresponding risk model. Similarly, the prediction efficiency of the risk score system was evaluated in different subtypes of cervical cancer.

### Nomogram Construction and Validation

Prognostic factors such as age, clinical stage, histological grade, pathological types, and risk score were used for the selection of the prognostic parameters to construct the nomogram through LASSO. The nomogram was constructed to assess the probability of 1-, 3-, 5- and 10-year overall survival with calibration curves for CESC patients using the “rms” R package. Then, the performance of the nomogram was visualized by AUC, concordance index, and DCA results.

### Evaluation of Immune Status Between Low- and High-Risk Groups

To investigate the relationships between the immune system and the prognostic signature, we further analysed the immune status between patients in low-risk group and high-risk group. The immune activity between the two groups was quantified based on single-sample gene set enrichment analysis (ssGSEA) by exploring the signatures of immune-related gene. Then, the relationships between the risk score and immune cells or relationships between the risk group and immune gene set expression level were further analysed.

### Immunotherapy Responsiveness Evaluation of Risk Signature and Related Genes

The Tumor Immune Dysfunction and Exclusion web (TIDE, http://tide.dfci.harvard.edu/login/) was used to predict the immunotherapy responsiveness of risk signature and related genes. TIDE is based on tumor pre-treatment expression profiles and can be used to estimate multiple published transcriptomic biomarkers to predict patient response. The published biomarkers (TIDE, MSI.score, TMB, CD274, CD8, IFGN, T.clonality, B.clonality, Merk18) based on their predictive power of response outcome were used for comparison.

### Clinical Benefits Evaluation of CESC OS Signatures

Recent years the net reclassification index (NRI), integrated discrimination improvement (IDI) have become the two popularity alternatives assessment in risk evaluation or prediction between different models (21, 22). We searched ten recently published CESC prognostic signatures, and used NRI, IDI method in “survIDINRI” package to compare the clinical benefits of our signature and published signatures (23–32).

## Results

### Acquisition of HLA-G-Driven DEGs in Cervical Cancer

**Figure 2:**
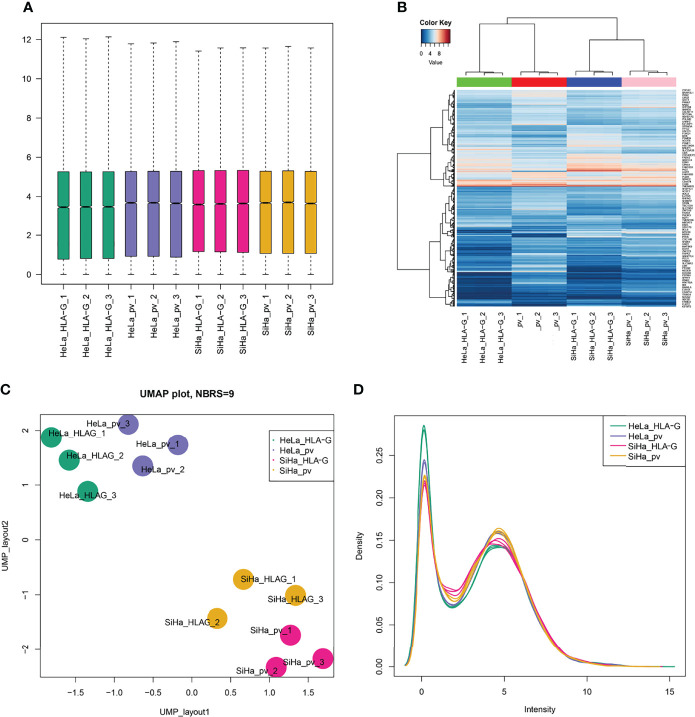
Read quality statistics after filtering by RNA-seq analysis. **(A)** The gene expression normalization of SiHa-pVITRO2-mcs, SiHa-HLA-G-pVITRO2-mcs, HeLa-pVITRO2-mcs and HeLa-HLA-G-pVITRO2-mcs cells after being sequenced by the BGISEQ-500 platform and quantified by the RSEM software package. **(B)**Heatmaps draw by “pheatmap” of 1083 genes that significantly co-expressed with HLA-G in SiHa and HeLa cells. **(C)** UMAP plotting of SiHa-pVITRO2-mcs, SiHa-HLA-G-pVITRO2-mcs, HeLa-pVITRO2-mcs and HeLa-HLA-G-pVITRO2-mcs cells with 1083 genes that significantly co-expressed with HLA-G. **(D)** The expression values density of SiHa-pVITRO2-mcs, SiHa-HLA-G-pVITRO2-mcs, HeLa-pVITRO2-mcs and HeLa-HLA-G-pVITRO2-mcs by “plotDensities” function in “limma” package.

**Figure 3:**
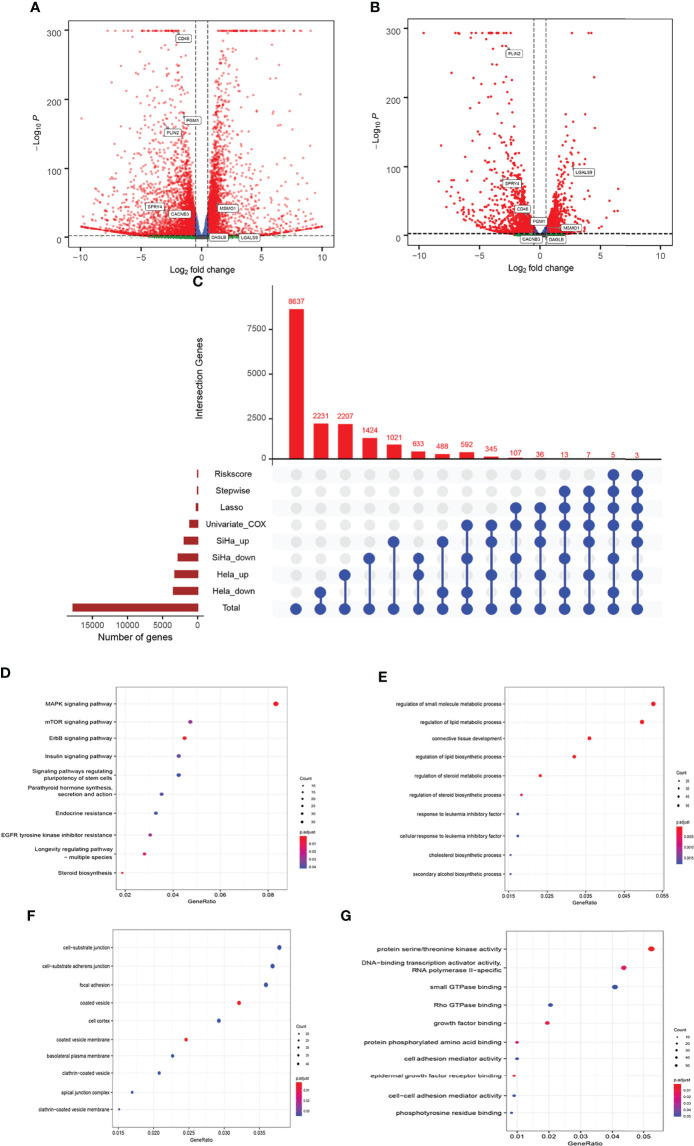
Comparison of gene expression profile which HLA-G induced of cervical cancer. Volcano of significantly DEGs in SiHa **(A)** and HeLa **(B)**, **(C)** Venn diagram, **(D)** the bubble plot of KEGG analysis, **(E–G)** the bubble plot of GO functional enrichment analysis. BP: Biological Precess. CC, Cellular Components. MF, Molecular Function.

In this study, HLA-G-driven DEGs were screened out by comparing different gene expression profiles of SiHa-HLA-G-pVITRO2-mcs versus SiHa-pVITRO2-mcs cells and HeLa-HLA-G-pVITRO2-mcs versus HeLa-pVITRO2-mcs cells (**Supplementary Figure 1**). Read quality statistics after filtering by RNA-seq analysis are shown in **Figure 2**. After normalization (**Figure 2A**), the expression level of 1108 HLA-G-driven DEGs, as shown in heatmaps, had clear clusters within different experimental groups (**Figure 2B**). UMAP a non-linear dimension reduction tool uses a “force-based layout” algorithm to built a weighted graph represent data at low dimensions (33). The UMAP analysis of the resultant total expression of different cell groups revealed clear spatial partitioning of each cell group (**Figure 2C**). The density plot also showed the difference expression value distribution of SiHa and HeLa cells with or without overexpression of HLA-G (**Figure 2D**). We identified 1900 upregulated and 2774 downregulated genes in the SiHa group (**Figures 3A, C**) and 3231 upregulated and 3436 downregulated genes in the HeLa group (**Figures 3B, C**), with thresholds of |Log_2_ FC| > 0.5 and *p*-value < 0.001. Finally, we selected the overlapping genes in the upregulated group and downregulated group, including 391 upregulated genes and 717 downregulated genes, which were identified as HLA-G-driven DEGs in cervical cancer (**Figures 3A–C**).

### Functional Annotations of HLA-G-Driven DEGs

The 1108 HLA-G-driven DEGs were mainly enriched in 559 GO terms and 68 KEGG pathways according to the functional enrichment analysis (*p*-adjust < 0.05). The KEGG results showed that the top five significantly enriched pathways were ErbB signalling pathway, steroid biosynthesis, MAPK signalling pathway, longevity regulating pathway-multiple species, and EGFR tyrosine kinase inhibitor resistance (**Figure 3D**). Meanwhile, the results indicated by GO enrichment showed that the top five significantly enriched terms in the biological process (BP) group were regulation of small molecule metabolic process, regulation of lipid metabolic process, connective tissue development, regulation of lipid biosynthetic process, and regulation of steroid metabolic process. In the cellular component (CC) group, the top five significantly enriched terms were coated vesicle membrane, coated vesicle, cell-substrate junction, cell- substrate adherens junction, and clathrin-coated vesicle membrane. In the molecular function (MF) group, the top five significantly enriched terms were protein serine/threonine kinase activity, DNA-binding transcription activator activity, RNA polymerase II-specific, epidermal growth factor receptor binding, growth factor binding, and protein phosphorylated amino acid binding (**Figures 3E–G**). These functional annotations suggested possible pathways or functional imbalances in which HLA-G-driven DEGs might be involved in the process of cervical cancer.

### Construction of PPI Network

**Figure 4:**
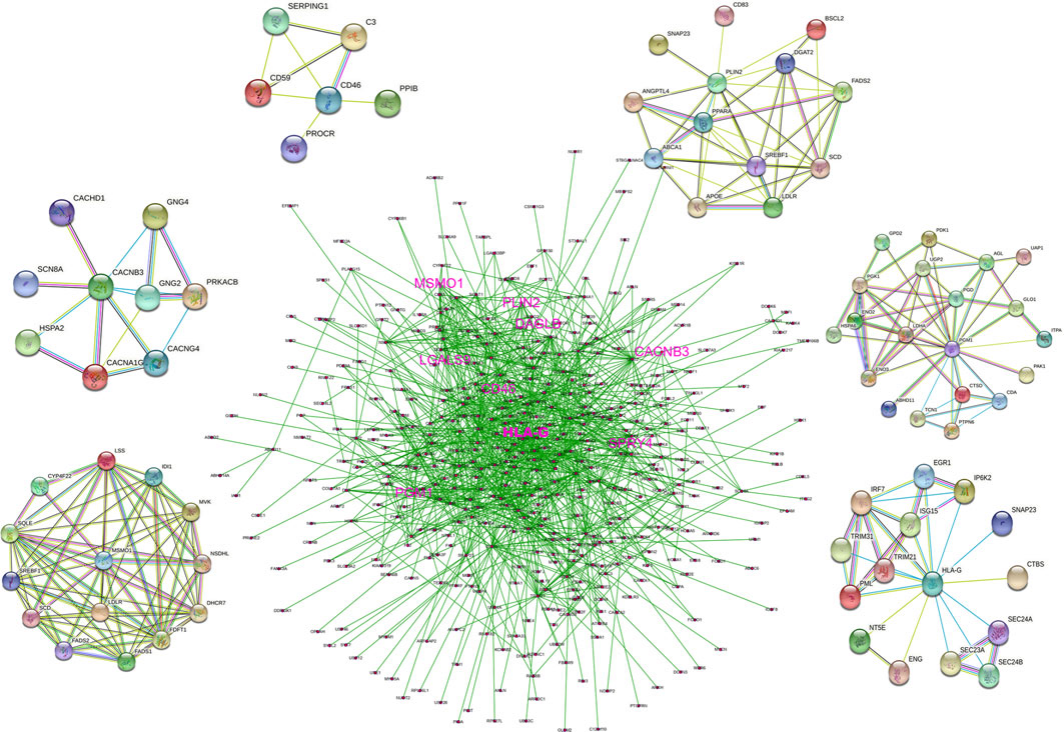
PPI network based on the data from STRING database.

We constructed a PPI network that incorporated 1097 nodes and 4072 edges based on the data from the STRING database (PPI enrichment *p*-value < 0.0001). The co-expression network was processed *via* Cytoscape software to identify possible key modules, and 375 genes that were directly or indirectly related to HLA-G and model-related genes were retained. A denser PPI network containing 375 nodes (34.2% of total) and 3062 edges (75.2% of total) is shown in **Figure 4**. *HLA-G*, *CD46*, *PGM1*, *CACNB3*, *PLIN2*, and *MSMO1* directly interacted with several genes, which indicated that the model-building genes may involve various biological functions.

### Construction of a Prognostic Model Based on HLA-G-Driven DEGs

**Figure 5:**
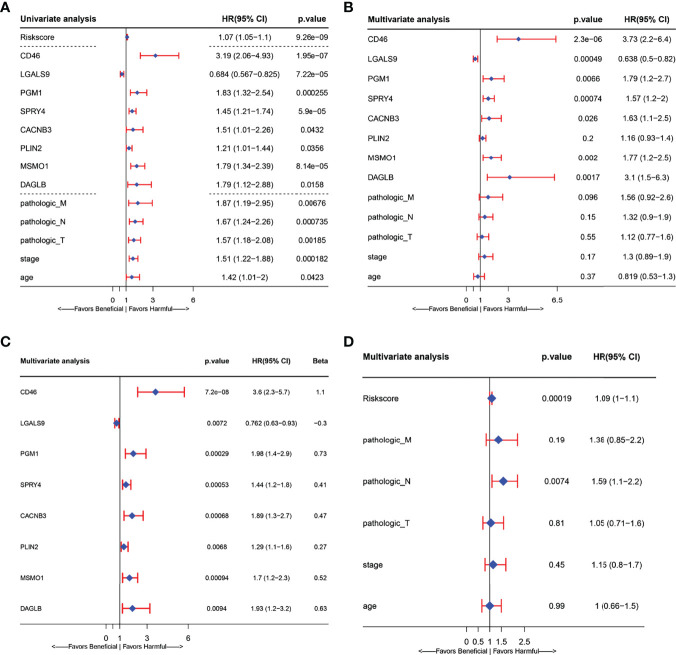
Forest plots presenting the univariate and multivariate Cox regression analysis of prognostic HLA-G-driven DEGs in model for overall survival (OS). **(A)** Forest plot for the univariate Cox analysis of risk score, risk signature related genes(CD46, LGALS9, PGM1, SPRY4, CACNB3, PLIN2, MSMO1, and DAGLB) and other clinicopathological factors: Pathologic_M, Pathologic_N, Pathologic_T, Stage and age in overall survival (OS) of cervical Cancer. **(B)** Forest plot for the relationship between risk signature related genes (CD46, LGALS9, PGM1, SPRY4, CACNB3, PLIN2, MSMO1, DAGLB) and other clinicopathological factors: Pathologic_M, Pathologic_N, Pathologic_T, Stage age in overall survival (OS) prediction of cervical Cancer. **(C)** Forest plot for the multivariate Cox analysis of signature related related genes in overall survival (OS) prediction of cervical Cancer. **(D)** Forest plot for the multivariate Cox analysis of risk signature and other clinicopathological factors: Pathologic_M, Pathologic_N, Pathologic_T, Stage age in overall survival (OS) prediction of cervical Cancer. (CI, confidence interval; HR, hazard ratio).

In a biological context, LASSO is widely used for gene expression and genomic data variable selection to identify a parsimonious model—a minimal subset of variables required to explain the data. There are relatively a few detailed reports of prognostic models based on gene expression profiles in cervical cancer (26, 28–30, 32). In this study, we selected the CESC prognostic markers from HLA-G driven DEGs buy LASSO regression. First, we integrated clinical data such as survival status, follow-up data, and clinicopathological information and matched them with the mRNA expression profiles of patients in the TCGA-CESC dataset. A total of 275 patients in the TCGA-CESC dataset with complete overall survival data. A total of 171 genes related to overall survival were screened in the TCGA-CESC cohort by univariate Cox regression analysis. Twenty-eight genes selected from LASSO (**Supplementary Figure 2**) were then used to identify key predictors of HLA-G-driven DEGs in the prognostic model for predicting the overall survival of cervical cancer patients by stepwise multivariate Cox regression. Then, eight signature-related genes are shown in the forest plots of **Figure 5**, connecting HLA-G-driven DEGs to *CD46, LGALS9, PGM1, SPRY4, CACNB3, PLIN2, MSMO1* and *DAGLB* in the OS model. These eight genes all showed a significant relationship with overall survival in both univariate (**Figure 5A**) and multivariate (**Figure 5B**) Cox regression analyses. Among them, only high expression of LGALS9 was related to a favourable prognosis in patients of cervical cancer (*P*=0.0072). According to the coefficient of each gene in the stepwise multivariate Cox (**Figure 5C** Beta), the risk score for this prognostic OS model=(1.10*expression level of CD46) + (-0.30*expression level of LGALS9) + (0.73*expression level of PGM1) + (0.41*expression level of SPRY4) + (0.47*expression level of CACNB3) + (0.27*expression level of PLIN2) + (0.52*expression level of MSMO1) + (0.63*expression level of DAGLB). As depicted in **Figure 5D**, we observed a significant correlation between the risk score signature and overall survival (*P*<0.001), which indicated that the risk score of this prognostic model could be used as one of the independent risk factor for cervical cancer patients. The KM curve and ROC curve of risk-related genes were also analysed. As shown in **Supplementary Figure 3**, the *p*-value of KM for all genes was less than 0.05. CD46, PGM1, DAGLB, and SPRY4 had relatively large AUC values for the ROC curves.

### Application and Validation of Prognostic Risk Model

**Figure 6:**
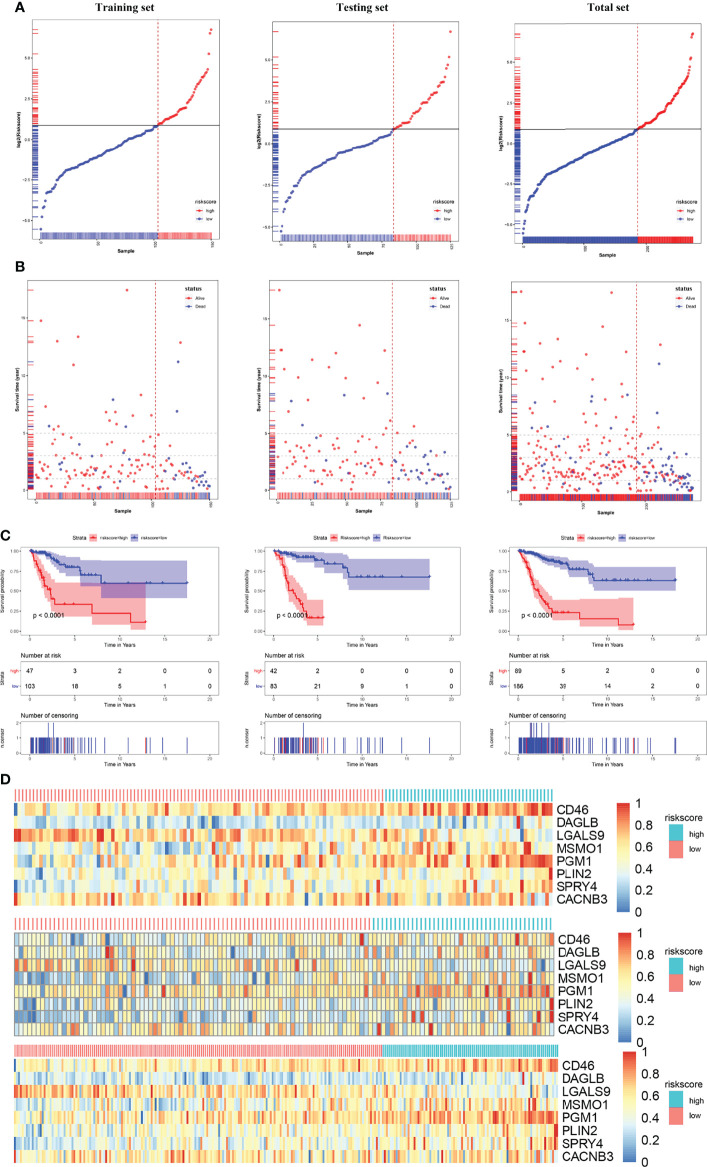
Risk score analysis, Kaplan-Meier analysis for the validation of prognostic model in training set, testing set and entire set. **(A)** Rank of risk score and distribution of groups. Patients were divided into low-risk group and high-risk group based on the median value of the risk score calculated. **(B)** The survival status and survival time of patients ranked by risk score. **(C)** Kaplan-Meier suggested that high-risk score group had shorter overall survival than low-risk score group in TCGA-CESC cohort. **(D)** The heatmap of the expression of eight HLA-G-driven DEGs in the signature in low- and high-risk score groups.

**Figure 7:**
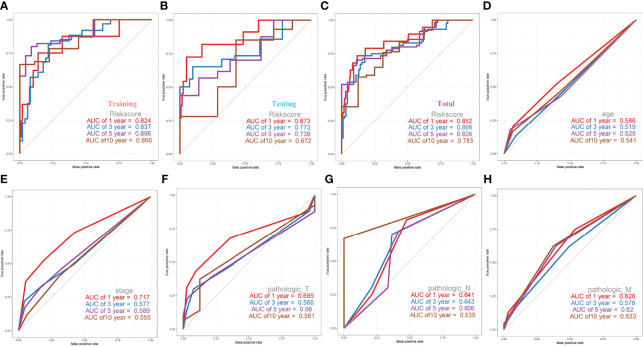
ROC Curves of OS for this prognostic model. **(A)** training set, **(B)** testing set, **(C)** entire set, **(D)** age, **(E)** stage, **(F)** pathologic_T, **(G)** pathologic_N, **(H)** pathologic_M.

After we obtained a relatively fixed cut-off value of the risk score by the “Surv_cutpoint” function through the training set to evaluate the testing set and the entire set. According to the fixed risk score cut-off value of 1.844096, patients were divided into low-risk scores group and high-risk scores group. The risk score distribution of each patient showed a high correlation with each patient’s survival status in the TCGA-CESC dataset, as shown in the dot plot figure (**Figures 6A, B**). Kaplan-Meier survival analysis showed that the OS time of the low-risk scores group was significantly longer than that of the high-risk scores group. The 5-year overall survival rates were 84.6% and 23.0% for the low- and high-risk scores patients, respectively (**Figure 6C**). The relationship between the relative expression level of eight signature-related genes and the relative risk score groups was shown in the heatmap (**Figure 6D**). In addition, the ROC curves based on the risk score were plotted, and the AUC signatures were 0.824 at 1 year, 0.837 at 3 years, 0.896 at 5 years, and 0.866 at 10 years (**Figure 7A**). We observed that the risk score signature had better predictive ability than other clinical factors (age, stage, pathological types, etc.) at 1, 3, 5, and 10 years (**Figures 7D–H**). In addition to the training set, we used the testing set and entire set of data to validate the prognostic value of this risk model. The results of the ROC curves in both the testing set and entire set were similar to those in the training set (**Figures 7B, C**), which indicated that the risk score signature has better sensitivity and specificity. In addition to the ROC curve, Kaplan-Meier survival subgroup analysis further validated the value of prognostic risk models in age (<45, 45-69 and >69), stage (stage I, II, III, IV) or pathological type (T, N, M).

**Figure 8:**
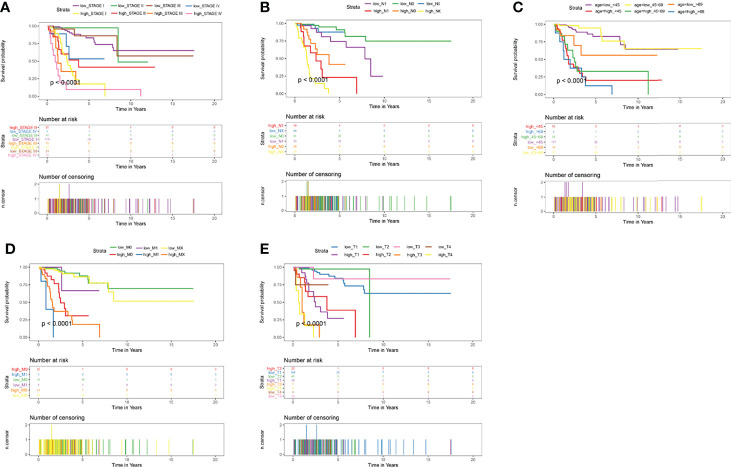
Kaplan-Meier Curves of OS for this prognostic model. **(A)** age, **(B)** stage, **(C)** pathologic_T, **(D)** pathologic_N, **(E)** pathologic_M.

Cervical cancer staging classification can stratify patient survival and help guide management more accurately. Multiple validation trials have demonstrated that stratified management of patients improves 5-year overall and disease-free survival. Given that stage T, N, M and ages were predictors of prognosis in cervical cancer patients, we compared the prognostic performance of these clinicopathological features with risk groups basted on our signature by survival curves. As depicted in **Figure 8**, the low risk patients owned relative long OS compared with high risk patients in the same clinicopathological grouping. In stage, Stage IV patients in low risk groups showed little difference OS with Stage I patients in high risk (**Figure 8A**). Similar significant different results of high and low risk groups were also found in different pathological M, pathological N, stage and age (**Figures 8B-D**). In pathological T, only T4 showed no relative significance, it may be because the patient’s T4 stage has too much influence on the patient, or the number of patients in the included T4 stage is too small (**Figure 8E**). Both these Kaplan-Meier curves and ROC curves suggested that this prognostic risk model constructed in this study could be used as a valuable prognostic tool to predict the OS of cervical cancer patients.

### Validation of the Prognostic Model in Subtypes of Cervical Cancer

**Figure 9:**
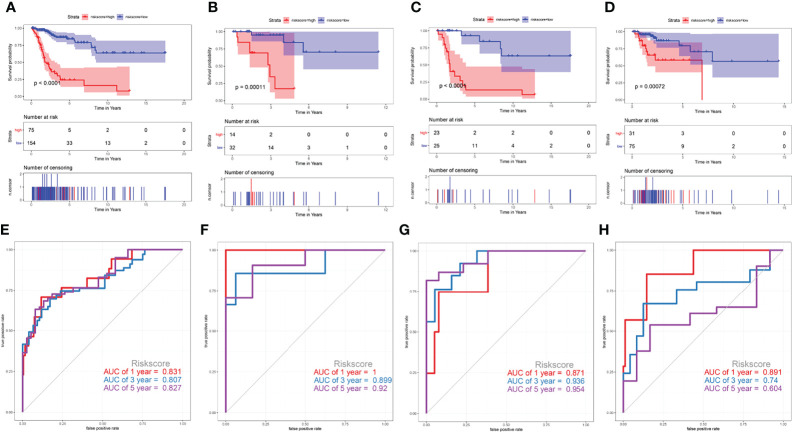
Validate the prediction efficiency of the risk score signature in different subtypes of cervical cancer. **(A, E)** squamous cell neoplasms, **(B, F)** non-squamous cell neoplasms, **(C, G)** keratinizing squamous cell carcinoma, **(D, H)** non-keratinizing squamous cell carcinoma.

To validate the prediction efficiency of the risk score signature of this corresponding risk model in different subtypes of cervical cancer. This prognostic model was also successfully validated in squamous cell neoplasms, non-squamous cell neoplasms, keratinizing SCC, and non-keratinizing SCC (**Figure 9**). The results of Kaplan-Meier analysis showed that the overall survival time of CESC patients with low-risk scores was significantly longer than that of patients with high-risk scores in those subtypes (*P*<0.001) (**Figures 9A–D**). The AUC values at 5 years were 0.827 for squamous cell neoplasms (**Figure 9E**), 0.92 for non-squamous cell neoplasms (**Figure 9F**), 0.954 for keratinizing squamous cell carcinoma (**Figure 9G**), and 0.604 for non-keratinizing squamous cell carcinoma (**Figure 9H**). These results indicate that this prediction model has a certain predictive effect on most CESC tumour types.

### Construction of a Nomogram Based on the Eight Genes

**Figure 10:**
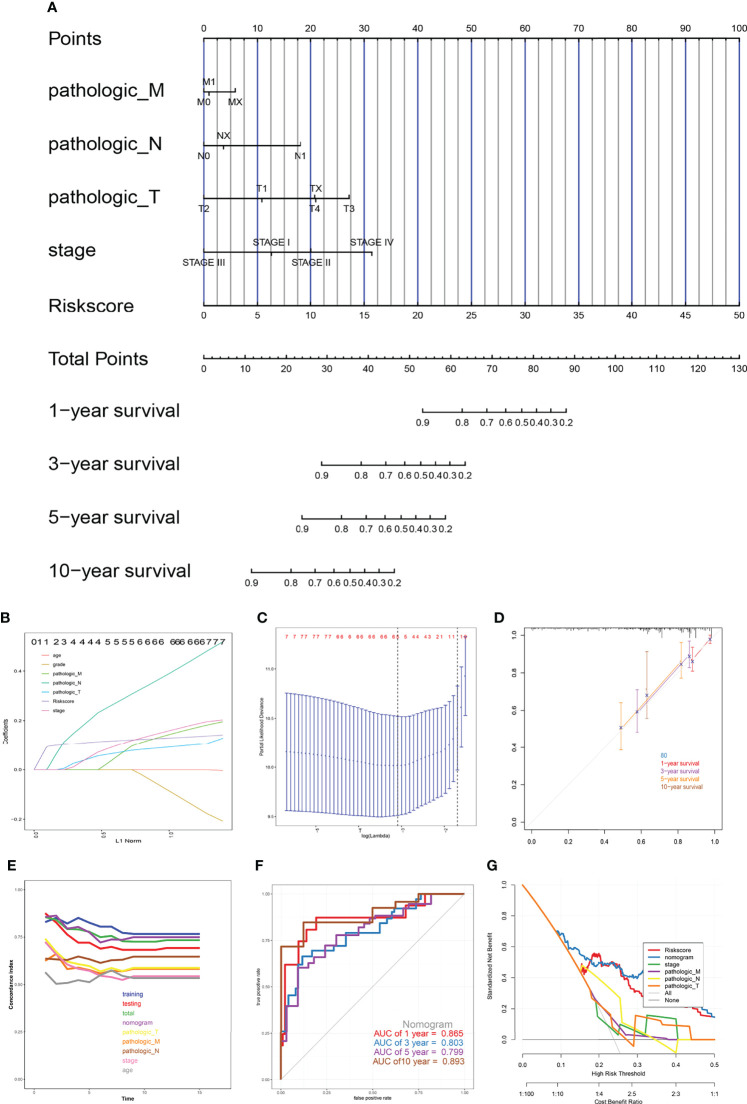
The nomogram model. **(A)** Prognostic nomogram for patients with cervical cancer, **(B, C)** Construction of prognostic nomogram models, **(D)** Calibration curves, **(E)** The calculation of the C-indexes in nine models, **(F)** ROC curve of overall survival for eight genes signature and clinical parameters, **(G)** Decision curve analysis.

After LASSO regression was used to test the significance of those traditional clinical prognostic parameters (age, sex, stage, grade, and pathological types) with the risk score signature (**Figures 10B, C**). A quantitative nomogram for CESC prognosis integrated the eight-gene signature, and the clinical prognostic parameters were established (**Figure 10A**), integrating the pathological TNM, stage, and risk score based on the LASSO results. The calibration curves after patients grouped into intervals containing 80 subjects on average were plotted to validate the nomogram performance. In the calibration plots, the predictive curves were close to the ideal curve for 1-, 3-, 5- and 10-year OS, indicating good performance (**Figure 10D**). Moreover, according to the C-index plot, at all inspection times, the nomogram curve was above the curve of tumour pathological TNM stage at all times. During most of the testing time, the nomogram curve was above the curve of the risk score in the total TCGA cohort (**Figure 10E**). These results combined with the ROC results (**Figure 10F**) indicated that the nomogram predicted survival better than a single prognostic factor. The DCA results showed that in a large risk threshold range, the nomogram and the risk score curve had higher benefits than the extreme curve, so their optional risk threshold ranges were relatively large and relatively safe (**Figure 10G**). This means that the the constructed nomogram has more clinical usefulness in predicting survival probability of cervical cancer patients. If the threshold probability was above 10%, using this nomogram to predict prognosis added more benefit than either the treat-none scheme or the treat-all scheme.

### Evaluation of the Relationship Between the HLA-G-Driven DEGs Signature and Immune Status in CESC

**Figure 11:**
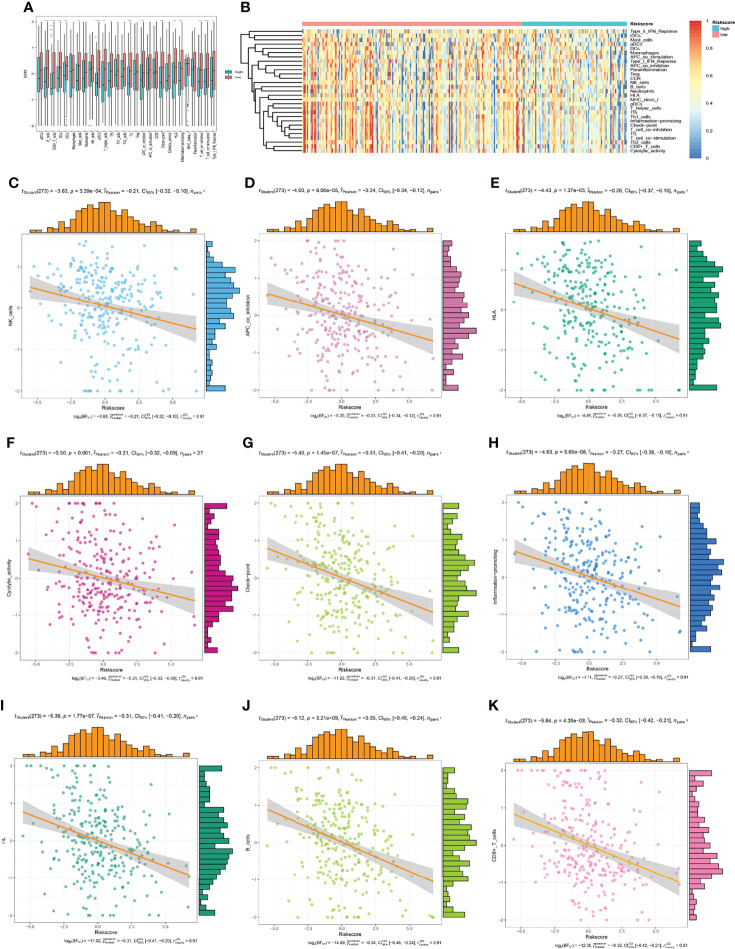
The low-risk group and high-risk group showed different immune status. **(A, B)** the expression profiles of the 29 immune-related gene sets, **(C–K)** the immune status of each patient in the low-risk group and high-risk group.

Immune infiltration in tumour microenvironment plays a key role in tumour progression and disease prognosis. We investigated the expression profiles of the 29 immune-related gene sets in the TCGA-CESC cohort. As depicted in **Figure 11A**, the low-risk group showed more immune activities, which could be caused by the higher infiltrated immune cells. Furthermore, the immune status of each patient in the low-risk group and high-risk group showed different degree of heterogeneity (**Figure 11B**). To understand the relationship between the risk model and the immune microenvironment more specifically, we analysed the risk score and the corresponding immune microenvironment score directly from each patient (**Figures 11C–K**). We found that the infiltration of CD8+ T cells, HLA, APC co-inhibition, and T cell co-stimulation was less in the high-risk group than in the low-risk group in the OS model. These results suggested that the eight genes in this prognostic model may play an important role in the immune regulation of the tumour microenvironment.

### Response Assessment of Signature Related Genes to Immune Checkpoint Blockade

**Figure 12:**
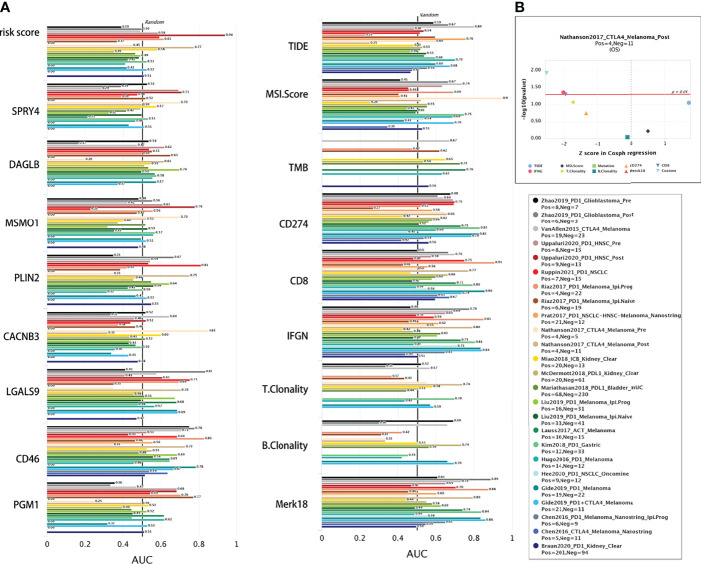
Comparison of immunotherapy response. **(A)** The immunotherapy response of risk signature and related genes were compared with the immune checkpoint blockade response status biomarkers by AUC in TIDE web. **(B)** The eight gene signature is association with overall survival of melamoma patients treated in CTLA4 (by two-sided Wald test).

The accuracy of the risk signature and eight signature related genes in comparison with other nine published immune checkpoint blockade response biomarkers were compared in different immunotherapy cohorts in TIDE web (**Figure 12**). Athough there is no data on cervical cancer, the signature gave an AUC greater than 0.5 in 10 out of the 21 cohorts (**Figure 12A**). The AUC of risk signature for PD1 response in head and neck squamous cell carcinoma is 0.94, which is larger than that of all other published markers. The AUC of the risk signature predicted the OS of melanoma treated with CTLA4 is 0.74. In addition, all these signature related genes also showed significant immune checkpoint blockade response in more than 9 cohorts (**Figure 12B**). The *p*-value of the signature prediction for the OS of melanoma treated with CTLA4 is less than 0.05. Above results suggesting that this signature and related genes may be robust predictive biomarkers for immune checkpoint blockade response.

### Prognostic-Related Gene Methylation Status of the Eight-Gene Signature

**Figure 13:**
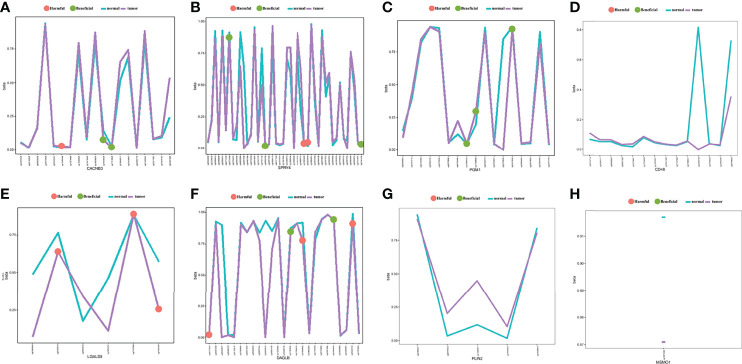
The methylation annotation file of the eight prognostic-related genes. Mean gene methylation level and the OS significance of eight risk signature genes **(A)** CACNB3, **(B)** SPRY4, **(C)** PGM1, **(D)** CD46, **(E)** LGALS9, **(F)** DAGLB, **(G)** PLIN2, and **(H)** MSMO1. (green: beneficial, red: harmful, turquoise: mean methylation level in normal, purple: mean methylation level in tumor sample).

**Figure 14:**
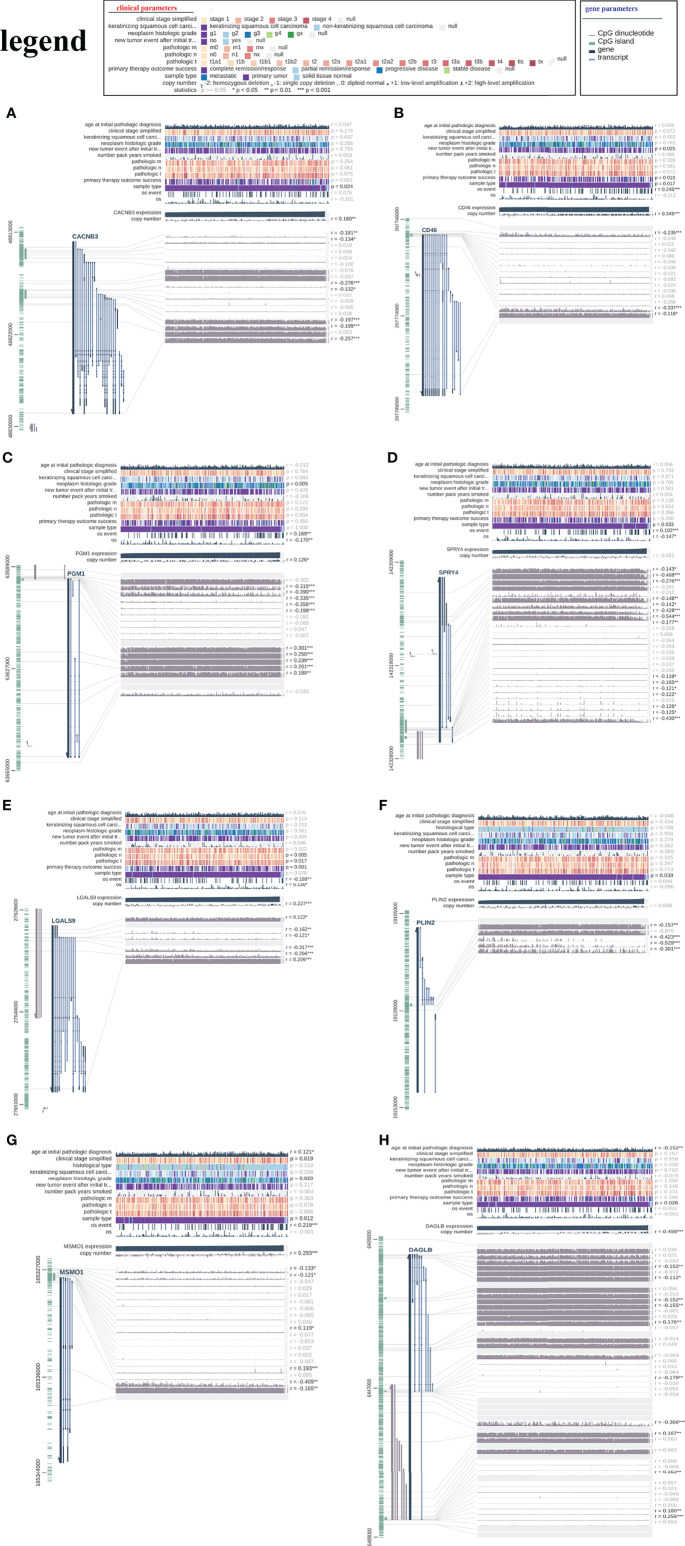
The relationship between the risk signature and DNA methylation status. The visualization of the relationships between TCGA expression, DNA methylation and clinical data by MEXPRESS for **(A)** CACNB3, **(B)** CD46, **(C)** PGM1, **(D)** SPRY4, **(E)** LGALS9, **(F)** PLIN2, **(G)** MSMO1 and **(H)** DAGLB.

We analysed DNA methylation data containing 309 CESC tissues and 3 normal tissues. The methylation β value of each methylation site is displayed in the scatter chart based on the arrangement of methylation sites. Through univariate Cox regression analysis, 10 beneficial sites and 9 harmful sites were collected from a total of 131 methylation sites of the eight related genes (**Figure 13**). PGM1 contained 3 beneficial sites, while LGALS9 had 3 harmful sites. PLIN2 and MSMO1 showed no significant sites for OS. In order to show the prognostic impact of these methylation sites, we performed survival status and clinicopathological information analysis. As depicted in **Figure 14**, the methylation sites of the *PGM1, SPRY4*, and *LGALS9* genes significantly correlated with the overall survival.

### The Eight-Gene Signature may Lead to Better Treatment Decisions for CESC Patients

Risk prediction have played important roles in making treatment decisions and improve treatment guidelines. Therefore, the accuracy of model predictions is crucial for beneficial treatment decisions. To further evaluate the predictive level of our model, we searched and compared 10 published cervical cancer prognostic signatures with our eight gene signature by NRI and IDI analysis. As results showed in **Supplementary Figure 4**, our eight gene signature performed better than the other then signature in 1, 3, 5 years OS prediction. but in ten year OS prediction, our signature showed less efficient than a seven gene signature (PLOD2, DSG2, SPP1, CXCL8, MCM5, HLTF, and KLF4) (32) and a six-gene signature (APOC1, GLTP, ISG20, SPP1, SLC24A3 and UPP1) (24). These results reveals that our signature may lead to a better treatment decisions comparison with other published prognostic signatures

## Discussions

According to the fact that HLA-G is a normal immune signal that can block the immune response, many recent studies have consistently claimed that HLA-G could be a new immune checkpoint molecule in tumours (8, 10, 11, 13, 34). HLA-G could inhibit almost all stages of the antitumour response by interacting with its inhibitory receptors ILT2 and ILT4 to transmit inhibitory signals. In addition, as a potential tumour marker, HLA-G can predict the diagnosis and prognosis of various types of cancer (7, 9, 11, 35). Therefore, exploring the relationship between HLA-G-positive tumours and their driving DEGs or their impact on the tumour microenvironment is very important for developing effective immunotherapy strategies. In our study, we established and validated, for the first time, a novel prognostic risk model for cervical cancer based on HLA-G-driven DEGs by using a series of bioinformatics methods. The eight HLA-G-driven DEG signatures of our prognostic model suggested great ability in predicting the overall survival of patients with cervical cancer. The prognostic value of the risk score signature was also evaluated in different subtypes of cervical cancer, which has been successfully verified in keratinizing SCC, non-keratinizing SCC, squamous cell neoplasms and non-squamous cell neoplasms. In addition, we established a nomogram model that integrated traditional clinical prognostic factors with the risk score signature and developed a quantitative method for CESC prognosis.

In this study, we obtained 1108 overlapping HLA-G-driven DEGs, which were subjected to GO and KEGG. The GO analysis suggested that HLA-G-driven DEGs were significantly enriched in coated vesicle membrane and cell-substrate adherens junction (in terms of CC group), regulation of lipid biosynthetic and metabolic process (in terms of BP group), protein serine/threonine kinase activity and epidermal growth factor receptor binding (in terms of MF group). These results suggested that HLA-G-driven DEGs were highly correlated with the cancer progression process (36–38). Additionally, KEGG suggested that HLA-G-driven DEGs were significantly enriched pathways in the ErbB, steroid biosynthesis, and MAPK, which are involved in cervical carcinogenesis (38–40). Previous studies have identified that somatic mutations of ErbB2 or ErbB3 receptors are significantly increased in cervical cancer, which provides potential therapeutic targets for patients (40, 41). After multivariate Cox regression analysis, eight genes were identified as key predictors of HLA-G-driven DEGs in the prognostic risk model for predicting the overall survival of patients with cervical cancer, including *CD46, LGALS9, PGM1, SPRY4, CACNB3, PLIN2, MSMO1*, and *DAGLB*.

Among eight genes in the prognostic model, CD46 was reported to be a key costimulatory molecule on CD4+ T lymphocytes and could facilitate the differentiation of CD4+ T cells into T regulatory 1 cells (Tr1 cells) secreting interleukin-10 (IL-10) and regulate the tumour immune microenvironment (TIME) (42, 43). The LGALS9 expression level was significantly correlated with HPV16 or HPV18 infection of cervical cells and showed a trend towards improved survival of patients (44, 45). SPRY4 is an inhibitor of the receptor-transduced MAPK signalling pathway, which plays a key role during carcinogenesis (46). CACNB3 may play a regulatory role in transcription factors or/and calcium transport (47). PGM1 was reported to be associated with glycogen metabolism (48). PLIN2 (perilipin 2) is a lipid storage-associated cell protein that is associated with the metabolism of intracellular lipid droplets (49). MSMO1 is believed to function in cholesterol biosynthesis (50). DAGLB is involved in proinflammatory signalling and the regulation of lipid pathways of the immune system (51). In the present study, the HLA-G-driven DEG signature suggested a great predictive ability in the OS of cancer patients.

Similar to HLA-G, CD46 was found to be upregulated in a variety of cancer cells (52). Consistent with previous data (53), our results suggested that high expression of CD46 was related to poor prognosis of cervical cancer. The AUC value at 5 years OS for CD46 was 0.729 (**Supplementary Figures S3A** and **3I**). Notably, CD46 expression in normal tissue is low, and it would be a suitable target for cancer immunotherapy (54). LGALS9 (also known as Galectin-9) is a β-galactoside binding protein that is the ligand for T cell immunoglobulin mucin-3 (TIM-3). Checkpoint molecule TIM-3 exerts immunosuppressive function by interacting with LGALS9, leading to immune escape during carcinogenesis (55, 56). Immunotherapy targeting the PD-1/PD-L1 has been successful in advanced cervical cancer, but the potential clinical value of other checkpoint molecules remains unknown, such as TIM-3/LGALS9 or HLA-G/ILT2. In this study, LGALS9 was a protective factor affecting the prognosis of patients, and its expression level was negatively associated with prognosis (*P*<0.001) (**Supplementary Figures S3B, J**). Notably, long noncoding RNA SPRY4-IT1 (SPRY4 intronic transcript 1) is a transcript variant derived from the second intron of the *SPRY4* gene, and it could be a good candidate biomarker for discriminating patients with cervical cancer in female population (57, 58). In addition, TIDE analysis showed that these genes also showed significant response in different immunotherapy treatments. Though the essential functions of the other six genes in cervical cancer remain unclear. Thus, further experimental validations of their functions in cervical cancer could strengthen our conclusion.

DNA methylation, the best characterized epigenetic alteration, the results in the exclusion of certain genes and plays an essential role in the progression of cancer (59). In this study, the lower expression of LGALS9 was related to the poor prognosis of cervical cancer patients in our prognostic OS model. A previous study found that this biological effect was mediated through the aberrant epigenetics of LGALS9, which was facilitated by the recruitment of DNMT3A to its promoter region (60). Using 5-aza-2’-deoxycytidine treatment, LGALS9 or SPRY4 expression can be affected by DNA methylation (60, 61). In recent years, DNA methylation changes have been utilized as biomarker for the early detection, diagnosis and prognosis of cervical cancer.

In addition, we explored the correlation between the risk model of cervical cancer and tumour-infiltrating immune cells. We compared the expression profiles of 29 immune-related gene sets between low-risk group and high-risk group, and explored their correlation with this prognostic model. The results suggested that there was less infiltration of CD8+ T cells and HLA in the high-risk group. A previous study showed that the overexpression of HLA-G molecules on the surface of tumour cells affects tumour-specific T cell immunity, and upregulate the expression of ILT2 on tumour-infiltrating CD8+ T cells in the cancer microenvironment (10). Therefore, blocking checkpoint molecule HLA-G could be used as a promising immunotherapeutic strategy for the treatment of cervical cancer.

## Conclusions

In summary, we established a novel prognostic risk model using HLA-G-driven DEGs through comprehensive bioinformatics analysis for the first time. These findings provide new insight into the biological markers, DNA methylation, and immunotherapy strategies in cervical cancer.

## Data Availability Statement

RNA sequencing data can be accessed via the GEO repository with accesison number GSE208119, access here: https://www.ncbi.nlm.nih.gov/geo/query/acc.cgi?acc=GSE208119.

## Author Contributions

H-HX wrote the first draft of the manuscript. All authors listed have made a substantial, direct and intellectual contribution to the work, and approved the submitted version.

## Funding

This work was supported by grants from National Natural Science Foundation of China (81901625), Natural Science Foundation of Zhejiang province (LY20H100004, LGF22H160058), Health Bureau of Zhejiang Province (2020KY1037).

## Conflict of Interest

The authors declare that the research was conducted in the absence of any commercial or financial relationships that could be construed as a potential conflict of interest.

## Publisher’s Note

All claims expressed in this article are solely those of the authors and do not necessarily represent those of their affiliated organizations, or those of the publisher, the editors and the reviewers. Any product that may be evaluated in this article, or claim that may be made by its manufacturer, is not guaranteed or endorsed by the publisher.

## Supplementary Material

Supplementary Figure 1 The protein expression of HLA-G in transfected and un-transfected cells by Flow Cytometry using HLA-G specific antibody MEM-G/09-FITC. **(A)** SiHa-pVITRO2-mcs. **(B)** SiHa-HLA-G-pVITRO2-mcs. **(C)** HeLa-pVITRO2-mcs. **(D)** HeLa-HLA-G-pVITRO2-mcs. MEM-G/09-FITC is an IgG1 antibody detecting native form of HLA-G on the cell surface.

Supplementary Figure 2 Establishment of prognostic risk model. **(A)** Tuning parameter (λ) selection in LASSO regression analysis. **(B)** LASSO coefficient profiles of 1108 HLA-G-driven DEGs.

Supplementary Figure 3 Kaplan-Meier and ROC of OS for genes in the signature. **(A, I)** CD46, **(B, J)** LGALS9, **(C, K)** PGM1, **(D, L)** SPRY4, **(E, M)** CACNB3, **(F, N)** PLIN2, **(G, O)** MSMO1 and **(H, P)** DAGLB.

Supplementary Figure 4 Clinical benefits evaluation by NRI and NRI analysis. Graphically display IDI, continuous NRI, and median improvement of eight gene signature compared with other 10 published CESC prediction signatures by IDI.INF.GRAPH in “survIDINRI” package.
