# Supplementary figures and images for: A Novel Prognostic Risk Model for Cervical Cancer Based on Immune Checkpoint HLA-G-Driven Differentially Expressed Genes
Source: Front Immunol. 2022 Jul 18;13:851622. doi: 10.3389/fimmu.2022.851622 (PMC9341272; doi:10.3389/fimmu.2022.851622)

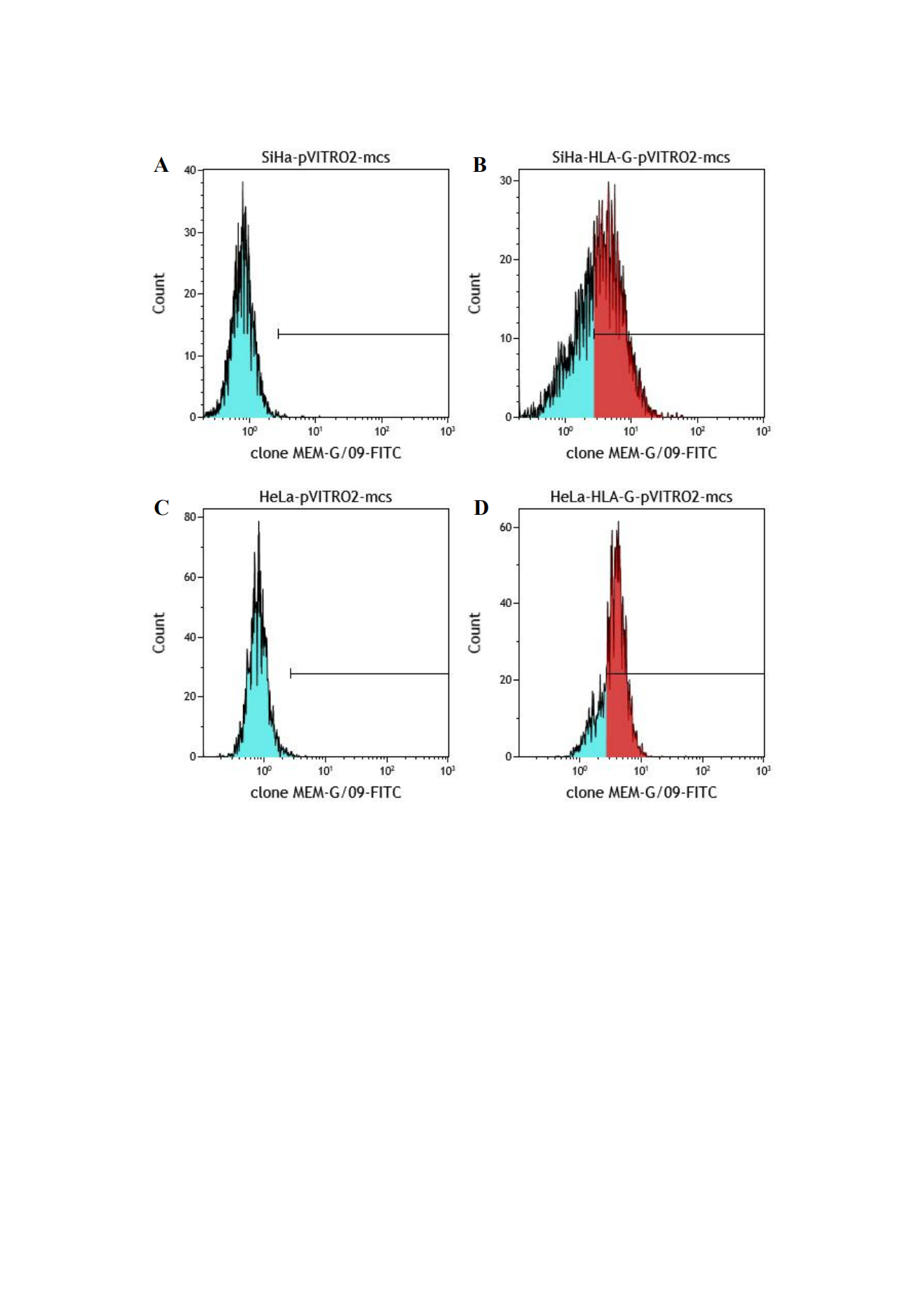

Supplement: Supplementary Figure 1 — The protein expression of HLA-G in transfected and un-transfected cells by Flow Cytometry using HLA-G specific antibody MEM-G/09-FITC. (A) SiHa-pVITRO2-mcs. (B) SiHa-HLA-G-pVITRO2-mcs. (C) HeLa-pVITRO2-mcs. (D) HeLa-HLA-G-pVITRO2-mcs. MEM-G/09-FITC is an IgG1 antibody detecting native form of HLA-G on the cell surface. [file Image_1.tif]

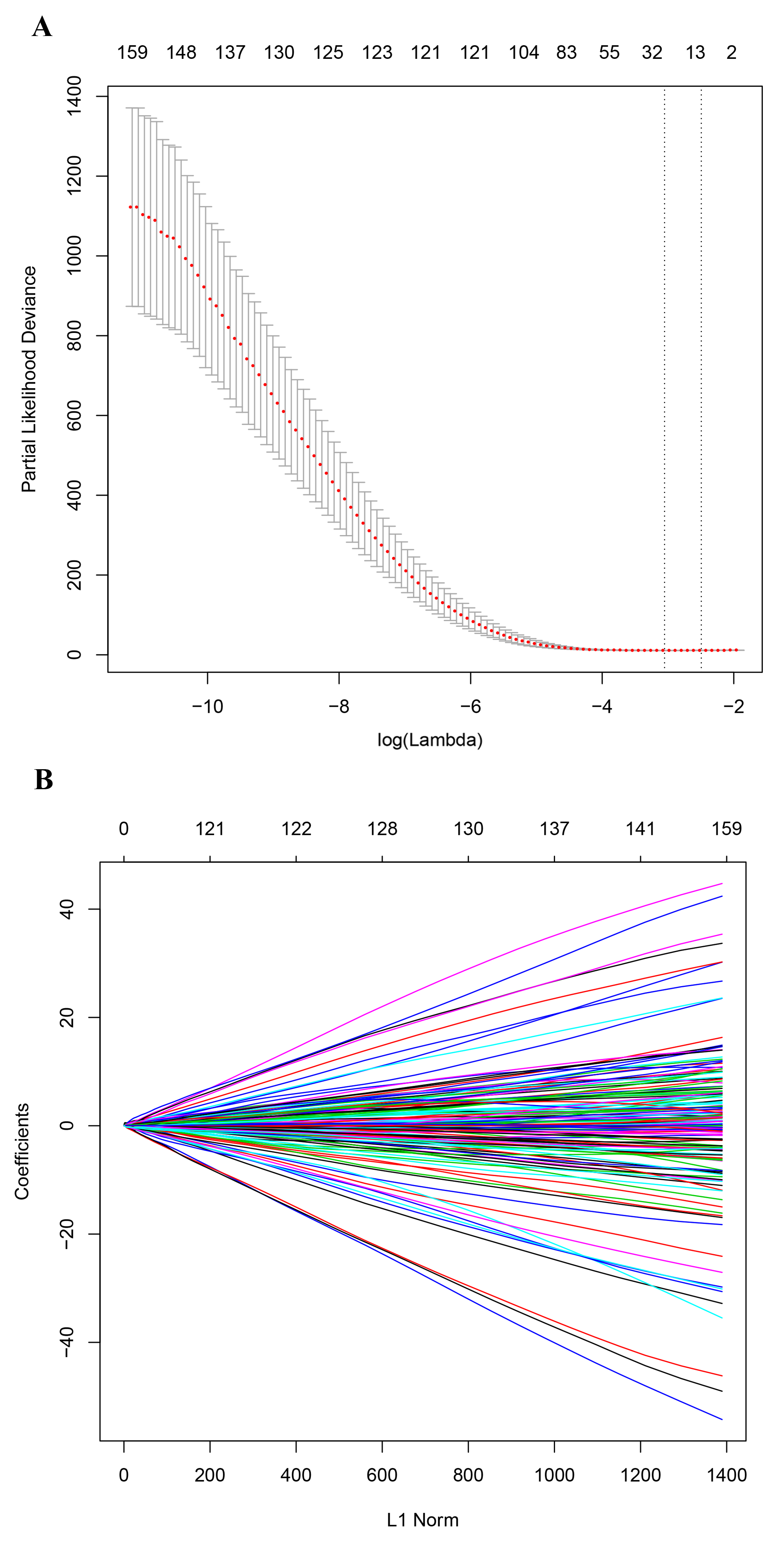

Supplement: Supplementary Figure 2 — Establishment of prognostic risk model. (A) Tuning parameter (λ) selection in LASSO regression analysis. (B) LASSO coefficient profiles of 1108 HLA-G-driven DEGs. [file Image_2.tif]

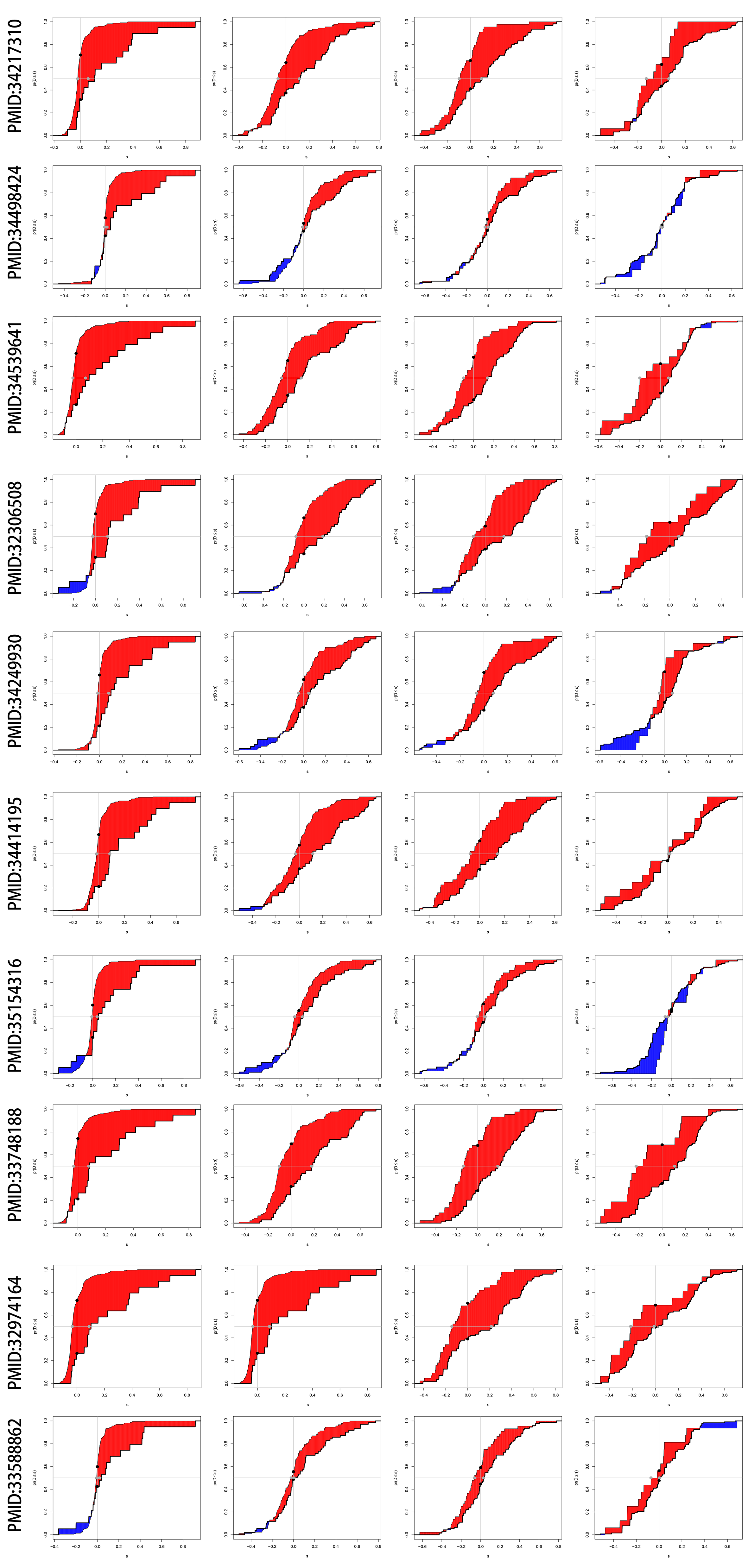

Supplement: Supplementary Figure 4 — Clinical benefits evaluation by NRI and NRI analysis. Graphically display IDI, continuous NRI, and median improvement of eight gene signature compared with other 10 published CESC prediction signatures by IDI.INF.GRAPH in “survIDINRI” package. [file Image_4.tif]
